# HIIT the Road Jack: An Exploratory Study on the Effects of an Acute Bout of Cardiovascular High-Intensity Interval Training on Piano Learning

**DOI:** 10.3389/fpsyg.2020.02154

**Published:** 2020-09-10

**Authors:** Dana Swarbrick, Alex Kiss, Sandra Trehub, Luc Tremblay, David Alter, Joyce L. Chen

**Affiliations:** ^1^Rehabilitation Sciences Institute, Faculty of Medicine, University of Toronto, Toronto, ON, Canada; ^2^Heart and Stroke Foundation Canadian Partnership for Stroke Recovery, Sunnybrook Research Institute, Toronto, ON, Canada; ^3^Hurvitz Brain Sciences Program, Sunnybrook Research Institute, Toronto, ON, Canada; ^4^RITMO Centre for Interdisciplinary Studies in Rhythm, Time and Motion, Department of Musicology, University of Oslo, Oslo, Norway; ^5^Department of Research Design and Biostatistics, Sunnybrook Research Institute, Toronto, ON, Canada; ^6^Department of Psychology, University of Toronto Mississauga, Mississauga, ON, Canada; ^7^Centre for Motor Control, Faculty of Kinesiology and Physical Education, University of Toronto, Toronto, ON, Canada; ^8^Department of Medicine, University Health Network, Toronto, ON, Canada; ^9^Institute of Health Policy, Management and Evaluation (IHPME), University of Toronto, Toronto, ON, Canada; ^10^Faculty of Medicine, University of Toronto, Toronto, ON, Canada; ^11^Cardiac Rehabilitation and Secondary Prevention Program, Toronto Rehabilitation Institute, University Health Network, Toronto, ON, Canada

**Keywords:** motor learning, music, cardiovascular exercise, sequence learning, transfer, consolidation, explicit

## Abstract

Pairing high-intensity interval training (HIIT) with motor skill acquisition may improve learning of some implicit motor sequences (albeit with some variability), but it is unclear if HIIT enhances *explicit* learning of motor sequences. We asked whether a single bout of HIIT after non-musicians learned to play a piano melody promoted better retention of the melody than low-intensity interval training (LIIT). Further, we investigated whether HIIT facilitated transfer of learning to a new melody. We generated individualized exercise protocols by having participants (*n* = 25) with little musical training undergo a graded maximal exercise test (GXT) to determine their cardiorespiratory fitness (VO_2__peak_) and maximum power output (W_max_). In a subsequent session, participants practiced a piano melody (skill acquisition) and were randomly assigned to a single bout of HIIT or LIIT. Retention of the piano melody was tested 1 hour, 1 day, and 1 week after skill acquisition. We also evaluated transfer to learning a new melody 1 week after acquisition. Pitch and rhythm accuracy were analyzed with linear mixed-effects modeling. HIIT did not enhance sequence-specific retention of pitch or rhythmic elements of the piano melody, but there was modest evidence that HIIT facilitated transfer to learning a new melody. We tentatively conclude that HIIT enhances explicit, task-general motor consolidation.

## Introduction

Excellence in music performance may require thousands of hours of practice (Ericsson et al., 1993; Freeman, 2000). Any means of improving the efficiency of such learning and other kinds of motor learning would be of enormous benefit. Recent research has demonstrated that some types of motor learning can be facilitated when paired with a single bout of high-intensity cardiovascular exercise (Roig et al., 2012; Mang et al., 2014, 2016b; Thomas et al., 2016a, b). The aim of the present study was to determine whether high-intensity cardiovascular exercise could also enhance learning a piano melody.

Cardiovascular exercise is beneficial for procedural (skill-based) long-term memory (Roig et al., 2013). The timing and intensity of the exercise is important, with the greatest benefits provided by higher intensities occurring in close temporal proximity, before and after motor learning (Thomas et al., 2016a, b). Specifically, high-intensity interval training (HIIT) is a cardiovascular exercise protocol that involves alternating intervals of high and low intensity (Tschakert and Hofmann, 2013). HIIT-induced increases in neuroplasticity may be the mechanism by which exercise promotes motor learning (Mang et al., 2014; Skriver et al., 2014; Taubert et al., 2015). HIIT alters the activity of the primary motor cortex (M1) by increasing intracortical facilitation and reducing inhibition, which may involve increases in LTP-like synaptic plasticity in M1 (Mang et al., 2014; Singh et al., 2014a, b). HIIT also reduces cerebellar inhibition, and *trans-*cerebellar sensory pathways may mediate the exercise-induced increases in M1 plasticity (Mang et al., 2014, 2016a). Lastly, exercise may increase M1 excitability through the release of neurochemicals including brain-derived neurotrophic factor (BDNF), dopamine, lactate, norepinephrine, epinephrine, and insulin-like growth factor (Quistorff et al., 2008; Skriver et al., 2014; Saucedo Marquez et al., 2015; Mang et al., 2017).

Previous research has investigated the role of a single bout of HIIT on motor learning and demonstrated that it promotes learning of several types of motor tasks (Mang et al., 2014, 2016b). These include continuous movement (no discernible beginning or end) (Lee and Genovese, 1989; Snow et al., 2016), discrete movement (clear initiation and end) (Lee and Genovese, 1989; Mang et al., 2016b), visuomotor tracking (Roig et al., 2012; Skriver et al., 2014; Thomas et al., 2016a, b; Lundbye-Jensen et al., 2017; Nepveu et al., 2017; Dal Maso et al., 2018; but see Hung et al., 2019 for a non-replication), and implicit motor-sequence learning (participants unaware of the sequence to be learned) (Mang et al., 2014; Song and Cohen, 2014), but not locomotor learning (Charalambous et al., 2018) or surgical skills (Chartrand et al., 2015). Much motor learning involves both implicit and explicit components (Krakauer et al., 2019). However, it remains unclear whether exercise promotes discrete, explicit motor-sequence learning, in which participants are aware of the motor sequence they have to learn. Specifically, no study has examined the effects of HIIT on musical sequence learning, which is arguably more complex than previously studied motor sequence learning because of the added constraints of memorization and timing. To our knowledge, moreover, no study has examined whether HIIT promotes transfer of learning to a new motor sequence.

Typical research studies on motor sequence learning involve the learner producing a sequence of movements that may or may not be cued. Learning a piano melody is a more complex form of motor sequence learning. In learning to play a melody, learners read a musical score or listen to an audio presentation, and from these sensory cues, learn to associate and produce key presses on the piano (Zatorre et al., 2007; Herholz and Zatorre, 2012). These discrete movements must also be executed accurately and expressively in pitch and time. Importantly, musicians often need to memorize what they perform, and they likely do so by creating an internal representation of the music, visually, aurally, or motorically (Godøy, 2010). Thus, learning to play a melody may be considered a complex motor learning task as it encompasses aspects of cognition, memory, and emotion (Serrien et al., 2007; Cisek and Kalaska, 2010).

Motor learning may be defined as relatively permanent changes to performance through experience (Schmidt et al., 2018). Motor learning involves three main phases: skill acquisition, consolidation, and retention. Acquisition is initial practice of a skill. Consolidation occurs after acquisition when memories transition from a fragile state to a more stable state (Krakauer and Shadmehr, 2006). During retention, a skill is tested either immediately after acquisition or at delayed time points (Salmoni et al., 1984). Motor skill consolidation relies in part on synaptic plasticity in the primary motor cortex (M1) (Muellbacher et al., 2002) and in part on systems consolidation across a number of networks including hippocampal and cerebellar regions (Dudai et al., 2015). Synaptic consolidation is especially important during early stages of motor consolidation and likely underpins systems consolidation (Dudai et al., 2015). Events that improve or degrade M1 plasticity after skill acquisition affect skill consolidation and thus retention (Attaway et al., 1999; Ramaekers et al., 2006; Borota et al., 2014; Greeley and Seidler, 2016).

The present exploratory study aimed to examine whether HIIT promotes consolidation and transfer of explicit motor sequence learning involved in piano learning. Non-musicians were trained to play a piano melody (skill acquisition) and were pseudo-randomized into a HIIT or low-intensity interval training (LIIT) group. Skill retention was tested 1 hour, 1 day, and 1 week after acquisition. Participants also practiced a new melody at 1 week to assess whether exercise-induced enhancements in initial skill consolidation promoted transfer of learning. We hypothesized that retention performance 1 day and 1 week after acquisition would be better for the HIIT group compared to the LIIT group. We also expected enhanced transfer to a new melody for the HIIT group.

## Materials and Methods

### Participants

Participants were blind to the hypothesis of the research and were recruited on the pretense that the purpose of the experiment was to understand the effects of exercise on motor learning. Participants were unaware of their group assignment and they were unaware of the other group. The participants (*n* = 25) were recruited with the inclusion criteria of being healthy, right-handed non-musicians between the ages of 18 and 35. For the present purposes, non-musicians were defined by having less than 5 years of musical training and no current musical practice (see Table 1). In Ontario, Canada where recruitment occurred, it is customary to have music classes that are mandatory in elementary and middle school, for as many as 5 years or more. We chose 5 years to be able to include those who had completed the mandatory training but to avoid recruiting anyone who had ever studied music as an elective. Exclusion criteria were previous participation in a video game competition, extensive experience in musical video games, any health condition [e.g., cardiovascular disease, neurological disorder, body mass index greater than 30 to approximate obesity status (World Health Organization, 2000), hearing or vision deficits that could not be corrected] or medications (e.g., antidepressants) that might affect the ability to perform the exercise or motor-learning elements of the study. We also screened for and excluded participants with amusia, a condition of impaired fine-tuned pitch discrimination, by assessing the ability to identify an instrumental version of a well-known song (Happy Birthday) (Ayotte et al., 2002; Peretz and Hyde, 2003). During data collection, two participants who met all inclusion/exclusion criteria were unable to complete the interval exercise test. Thus, we added the exclusion criterion of exercising less than once per week. All participants provided written informed consent prior to the first session. This study was approved by and carried out in accordance with the ethical standards of the University of Toronto Office of Research Ethics. All subjects gave written informed consent in accordance with the Declaration of Helsinki, 2013.

**TABLE 1:** Descriptive data of participants.

								GXT					IET	
Pt	Int	Sex	Age	Comp.	EFT	M.Train	IPAQ	VO_2__peak_	W_max_	HR_max_	RER_max_	RPE_max_	HR_max_	RPE_max_
1	Low	F	23	6	0	24	3106.5	47.2	230	182	1.12	17	100	7
2	Low	M	23	7	22	26	462	38.3	160	180	1.16	18	94	7
3	Low	F	21	5	16	12	1108.5	29.3	110	183	1.2	18	107	10
4	Low	F	25	3	18	1	537	28.9	110	165	1.04	15	107	8
5	Low	M	20	7	12	0.75	758	43.3	190	203	1.12	17	119	9
6	Low	F	24	2	6	24	132	26.9	110	140	1.38	18	122	10
7	Low	M	21	6	1	2	3804	31.9	220	181	1.15	19	105	14
8	Low	F	18	2	2	0	1320	24.8	80	146	1.19	16	105	10
9	Low	M	18	7	0	0	660	35.9	160	192	1.19	20	130	10
10	Low	F	20	5	0	12	702	23.5	80	186	1.19	18	132	9
11	Low	M	22	6	12	24	6984	44	220	193	1.17	16	97	9
12	Low	F	23	1	6	1.5	1986	22.9	80	177	1.13	17	113	11
13	High	M	24	5	0	58	798	41.6	220	196	1.17	19	183	16
14	High	F	30	7	3	12	3999	25.1	110	164	1.12	16	156	15
15	High	M	24	6	17	0.5	1155	24.6	100	171	1.27	15	179	18
16	High	F	21	5	3	36	660	19.8	80	189	1.18	17	193	18
17	High	F	23	4	13	0	66	27.8	110	173	1.18	18	173	19
18	High	F	19	6	7	5	693	27.6	110	178	1.14	17	181	17
19	High	F	24	6	5	2	4158	38.5	170	178	1.14	18	174	18
20	High	F	23	7	12	12	3132	28.7	140	189	1.21	19	181	20
21	High	F	20	7	0	18	330	25.1	110	182	1.11	17	172	16
22	High	F	18	3	0	0.75	912	22.4	110	182	1.13	20	177	13
23	High	M	18	7	4	1	1158	46	220	174	1.09	17	176	15
24	High	M	23	4	4	10	758	32.6	160	195	1.17	17	202	19
25	High	M	22	5	8	5	4158	36.9	190	179	1.25	19	170	16
M. Low	*n* = 12	M: 5; F: 7	21.5	4.8	7.9	10.6	1796.7	33.1	145.8	177.3	1.17	17.4	110.9	9.5
M. High	*n* = 13	M: 5; F: 8	22.2	5.5	5.8	12.3	1690.5	30.5	140.8	180.8	1.17	17.6	178.2	16.9

*Pt, Participant; Int, Intensity; Comp., Competitiveness; EFT, extra familiarization trials; M.Train, Months of Musical Training; IPAQ, International Physical Activity Questionnaire Totals (MET-minutes/week); GXT, Graded exercise test parameters; IET, interval exercise test parameters; VO_2peak_, Peak volume of oxygen consumed (mL/kg/min); W_max_, Maximal power output; HR_max_, Maximum heart rate (bpm); RER_max_, Maximum respiratory exchange ratio; RPE_max_, Maximum rating of perceived exertion (Borg 6–20 scale). Participants 16, 17, 20, 21, and 24 failed to complete their personalized high-intensity interval training protocol.*

### Study Design

**FIGURE 1:**
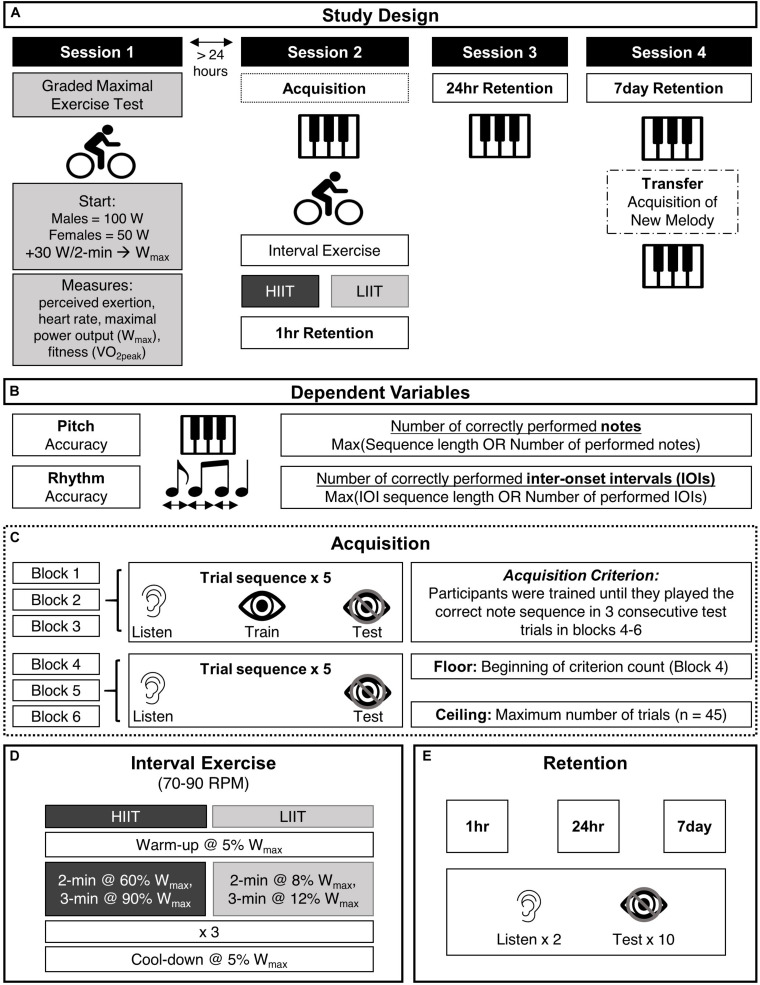
Methods. **(A)** The study consisted of four sessions (details are in the “Materials and Methods” section): During session 1, participants underwent a graded maximal exercise test. During session 2, participants practiced a melody on a piano keyboard (piano acquisition) and underwent either high- or low-intensity interval training (HIIT or LIIT) on a stationary cycle ergometer **(D)**. They completed retention tests 1-h (session 2), 24-h (session 3), and 7-days (session 4) after initial training. During session 4, they also practiced a new melody in the transfer task. **(B)** The dependent variables were pitch and rhythm accuracy. Pitch accuracy was measured as the number of correctly performed notes divided by the longer of either the sequence length or the number of performed notes. Rhythm accuracy was measured as the number of correctly performed inter-onset intervals divided by the longer of either the inter-onset interval sequence length or the number of performed inter-onset intervals. **(C)** The piano acquisition protocol consisted of six blocks. In blocks 1–3, participants completed five rounds of a trial where they listened to the melody (listen), a trial in which they played along with visual cues from a computer game and heard their auditory feedback (train), and a trial in which they performed the melody in the absence of visual cueing while hearing their auditory feedback (test). In blocks 4–6 participants had the opportunity to complete five rounds of a listen trial (hearing the melody) and a test trial (performing the melody without visual cueing while hearing their auditory feedback). However, once a participant reached the criterion of performing the pitch sequence correctly in three consecutive trials, the acquisition phase was concluded (See section “Trial Types” for more information). **(D)** Participants were randomized into either a high- or low-intensity interval training group (HIIT or LIIT). They cycled at a cadence between 70 and 90 revolutions per minute (RPM). They first began a warm-up and then each group alternated between their personalized high and low intensities. There was a cool-down after the exercise protocol. **(E)** Retention tests, conducted at 1-h, 24-h, and 7-days after acquisition, consisted of two listen trials and ten test trials.

Our study design was similar to that of previous studies demonstrating the effects of HIIT on motor learning (Roig et al., 2012; Mang et al., 2016a, b; Thomas et al., 2016a). In particular we modeled the exercise parameters after Mang et al. (2016a, b) who also recruited both men and women to maximize the comparability between our manipulation of motor learning task and previous research with positive findings. Participants attended four sessions in which they completed: (1) questionnaires, assessments, and a graded exercise test; (2) the skill acquisition phase of the piano melody, an interval exercise test (HIIT or LIIT), and 1-h retention test; (3) a 24-h retention test; and (4) a 7-day retention test and transfer test (Figure 1). After each session, participants were asked to report their strategies and thoughts on tasks completed that day.

#### Randomization

We matched participants according to gender and fitness (ascertained in session 1) for pseudo-randomization prior to the piano acquisition phase (session 2). In each matched pair, one person was assigned randomly to HIIT and the other to LIIT. Preliminary analysis revealed unexpected group differences in melody acquisition prior to the interval exercise test (HIIT or LIIT). As a result, participants with IDs 10, 11, 12, 24, and 25 were also randomized according to the number of familiarization trials required before the acquisition protocol.

#### Assessments and Questionnaires

We gathered demographic information and administered assessments and questionnaires to evaluate if there were baseline differences between groups. We assessed beat perception abilities, using five stimuli from the Beat Alignment Task (BAT) (Iversen and Patel, 2008). In this task, participants judged whether a superimposed track of isochronous beeps was ON or OFF the beat of the underlying music. Auditory working memory (AWM) was assessed with an auditory forward digit span task (Inquisit,© Millisecond Software). Questionnaires were used to gather information on musical experiences and preferences (Litle and Zuckerman, 1986; Rentfrow and Gosling, 2003; Iversen and Patel, 2008), competitiveness, physical activity habits (International Physical Activity Questionnaire; Craig et al., 2003), motivational state (Wulf and Lewthwaite, 2016), alertness (Hoddes et al., 1973; Borragán et al., 2016), and recent caffeine (Borota et al., 2014), nicotine, and food consumption. Participants also filled a daily log to track quantity and quality of sleep and exercise.

#### Graded Exercise Test

Participants performed a graded maximal exercise test (GXT) on a cycle ergometer. The GXT was used to determine cardiovascular fitness (VO_2peak_) and maximum power output (W_max_). The GXT protocol was based on (Mang et al., 2016a,b), which prescribes unique intensities for men and women. VO_2peak_ was used to ensure that there were no average fitness differences between groups and the measure of W_max_ was used to prescribe the personalized interval exercise protocol described in the section below.

Participants’ weight and height were measured, the height of the saddle and handlebar of the cycle ergometer (Ergomedic 839E, Monark, Sweden) were adjusted, and participants were fitted with a heart rate (HR) monitor (Polar H7) and mask attached to a metabolic cart (ParvoMedics TrueOne 2400, Sandy, UT, United States). HR, volume of consumed oxygen (VO_2_), and respiratory exchange ratio (RER) were monitored. Participants were instructed to remain seated throughout the test and to maintain a cycling cadence between 70 and 90 revolutions per minute (RPM). The cycle ergometer was electronically braked so that work rate was maintained regardless of the participants’ preferred cycle cadence between 70 and 90 RPM (the resistance automatically increased with lower cadence and decreased with higher cadence). They were instructed to continue as long as possible and to perform as well as they could. However, they were asked to stop if experiencing chest pain, dizziness, or faintness and to inform the experimenter immediately.

Men began the test at 100 W while women began at 50 W (Mang et al., 2016a, b). The power output was increased by 30 W every 2 min. In the middle of each 2-min interval, participants reported subjective exertion levels using Borg’s 6–20 rating of perceived exertion (RPE) scale (Borg and Kaijser, 2006). The test ended when participants reached volitional exhaustion or were unable to maintain a cycling cadence above 70 RPM despite verbal encouragement. To ascertain that participants reached their VO_2peak_, at least one of the following criteria had to be met: plateau in VO_2_ or HR with further increase in workload, RER > 1.1, inability to maintain the target cadence, or volitional exhaustion (Roig et al., 2012; Mang et al., 2014, 2016a; Skriver et al., 2014). Participants’ W_max_, defined as the power output during the final, fully completed stage of the GXT, was used to prescribe the personalized intensities for the interval exercise test (Mang et al., 2014).

#### Skill Acquisition of the Melody

**FIGURE 2:**
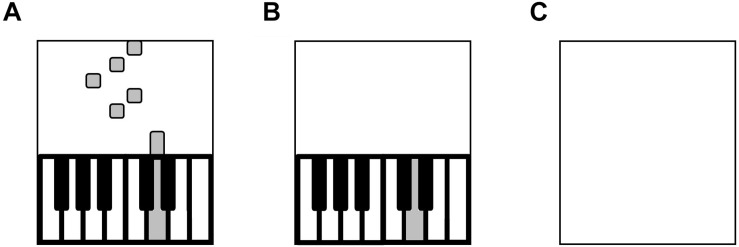
Trial Types. **(A)** Training trial with visual cues. **(B)** Test trial in blocks 1–3 without visual cueing. **(C)** Test trial in blocks 4–6 without on-screen keyboard.

Skill acquisition took place at least 24 h after the initial session (graded exercise test). Participants practiced the melody on a MIDI piano keyboard (Yamaha YPT-210) connected to a laptop computer (Asus, UX360UAK) via a USB MIDI Interface (UM-ONE, © Roland). Participants placed their right hand and fingers on five stickered keys and faced the laptop screen. We used Synthesia (©2018 Synthesia, LLC), a software program designed to teach piano playing using visual cues. Synthesia visualizes musical notes with rectangles that descend onto an on-screen piano. The length of each rectangle indicates the duration of the note and the location along the horizontal plane indicates the pitch of the note (see Figure 2A). Participants were instructed to press the corresponding piano key when the descending rectangle hit the on-screen piano.

##### Trial types

The piano acquisition task aimed to enable non-musicians to perform the melody from memory without visual cues. Pilot testing determined the type and number of trials required for skill acquisition. Three types of trials were presented: (1) listen, (2) training, and (3) test. During listen trials, participants were instructed to keep their fingers still and listen as the piano melody played (over loudspeakers) without accompanying visual cueing. These trials were designed to promote a mental auditory representation of the melody, so that participants could detect their own errors during training and test trials (McAdams, 1987; Padrão et al., 2014). During training trials, participants attempted to play and memorize the visually cued melody (Figure 2A), which was accompanied by auditory feedback of their performance, presented over the keyboard’s speakers. During test trials, participants attempted to play the melody from memory without visual cueing, but with auditory feedback of their performance (Figure 2B). Four metronome beats preceded the training and test trials to allow participants to internalize the beat and facilitate reproduction of the rhythm.

##### Familiarization

**FIGURE 3:**
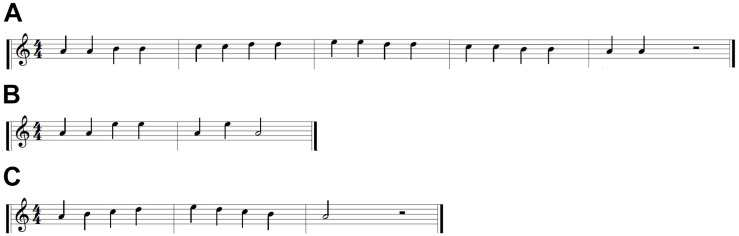
Familiarization melodies. **(A)** Melody 1. **(B)** Melody 2. **(C)** Melody 3.

Prior to practicing the target melody, participants were required to demonstrate comprehension of the task. They were familiarized with the trial types using three simple melodies (Figure 3). Once participants played each familiarization melody correctly twice in both training and test trial formats, they began the main piano acquisition task. The number of familiarization trials required to demonstrate comprehension was used as an estimate of baseline capabilities.

##### Blocks

Participants practiced six blocks of the target melody. Blocks 1–3 comprised five trials each of listen, training, and test, presented in that order, for a total of 15 trials per block (Figure 1) to facilitate memorization of the melody with visual cues. The on-screen piano key was illuminated in green when participants played a correct note at the correct time. When an incorrect note was played, or a correct note was played at an incorrect time (>200 ms early or late), the on-screen keyboard key was illuminated in gray. To minimize participants’ dependence on such feedback, blocks 4–6 consisted of five trials each of listen and test trials for a total of 10 trials per block, with the on-screen keyboard hidden from view (Figure 2C).

##### Criterion training

Because large inter-individual variability in performance became apparent during pilot testing, we instituted a protocol in which participants were trained to a specified criterion. Participants performed a minimum of blocks 1–3 and practiced until they reached a criterion of three consecutive correct test trials. A correct test trial was defined as the correct sequence of pitches without consideration of rhythm accuracy. Thus, once participants achieved three consecutive correct test trials, they stopped the skill acquisition phase and proceeded to the interval exercise test. If participants did not reach this criterion, they continued practicing until the end of block 6.

##### Stimuli

**FIGURE 4:**
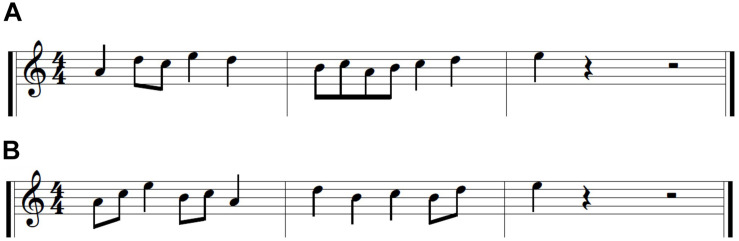
Piano melodies. **(A)** Melody 1. **(B)** Melody 2.

We selected two melodies approximately matched for difficulty in previous research (Brown and Penhune, 2018; see Figure 4). One melody was used for the acquisition phase, the other for the transfer phase. The two melodies consisted of 12 notes with five unique pitches (A4, B4, C5, D5, E5); their rhythms consisted of quarter notes and eighth notes at a tempo of 75 bpm. Participants used their right hand to perform the melodies, and each of the five unique pitches was assigned to a single digit.

The two melodies were counterbalanced across participants with the constraint that participants and their matched pairs (HIIT or LITT) were randomly assigned to one of two melodies. Thus, each pair played the same melody during acquisition and the other melody during transfer.

##### Task details

The laptop’s screen (UX360UAK, ASUSTek Computer Inc.), with a resolution of 1920 × 1080p, was adjusted so that the on-screen keyboard lined up with the actual MIDI piano keyboard. The experimenter provided a brief description of the experimental session. Pre-recorded instructions that described the piano learning task were presented over loudspeakers (MultiMedia Speaker Model A215, Samsung Electro-Mechanics Co., Ltd.). Musical stimuli were presented through the keyboard’s speakers at comfortable sound levels.

A custom Python script (version 3.6)^1^ automated the recorded instructions and the transitions between trials using several Python libraries including MIDO (version 1.2.8, © 2014 Bjørndalen, O., MIDI Objects for Python^2^), PyAutoGUI (version 0.9.36, © 2014, Sweigart, A^3^), and dill [version 0.2.7.1, (McKerns and Aivazis, 2010; McKerns et al., 2011)]^4^. Participants’ performance was recorded using a custom Python script that also used MIDO and dill.

#### Retention Tests

During the retention tests, participants performed two listen trials to cue their memory of the melody and 10 test trials, with a 4-beat metronome count-in and no visual cues. An immediate retention test took place 1 h after acquisition (R1h), approximately 20 min after the end of the interval exercise protocol. Delayed retention of skill learning was tested 24 h (±2 h) (R24h) and 7 days (R7d) (±2 h) after acquisition (Kantak and Winstein, 2012).

#### Interval Exercise Test

Participants were fitted with a HR monitor and the cycle ergometer was adjusted for comfort. Participants were instructed to maintain a cycling cadence between 70 and 90 RPM and to do their best to complete the interval exercise protocol. Borg’s ratings of perceived exertion were recorded in the middle of each interval (Borg and Kaijser, 2006).

The interval exercise test was 19 min in duration and the main interval exercise consisted of three repetitions of 2-min low-intensity intervals and 3-min high-intensity intervals. Intensities were prescribed based on each participants’ W_max_. The experimental (HIIT) group’s low intensity was 60% W_max_ and their high intensity was 90% W_max_ (Thomas et al., 2016a, b). This protocol was specifically selected to cause a substantial increase in blood lactate levels (Roig et al., 2012; Thomas et al., 2016a, b). The control (LIIT) group performed low-intensity intervals at 8% W_max_ and high-intensity intervals at 12% W_max_. There was a 2-min warm-up and a 2-min cool-down at 5% W_max_. The low intensities of 8% and 12% W_max_ were chosen to have the same ratio difference as the experimental group (2:3), and they were less likely to promote motor consolidation (Roig et al., 2013; Thomas et al., 2016b).

#### Transfer Test

Following the 7-day retention test, the participant completed the transfer test. Participants practiced playing a new melody, using the same acquisition protocol described in section “Skill Acquisition of the Melody” (Figure 1), but they continued to practice even if they reached the criterion of three consecutive correct trials. Thus, every participant practiced for the same number of trials.

### Data Analysis

Timing and pitch information were recorded from participant keypresses with custom Python scripts. Dependent measures were pitch and rhythm accuracy during test trials.

#### Pitch Accuracy

Pitch accuracy was defined as the percentage of correctly performed notes. Sub-sequences of the target sequence were identified in the performed sequence. If participants performed these sub-sequences in the correct order they were counted toward the total number of correctly performed notes. This total was divided by whichever was greater between the total number of notes in the sequence (*n* = 12) or, to penalize excessive notes, the number of performed notes.

#### Rhythm Accuracy

Rhythm accuracy was defined as the percentage of inter-onset intervals (IOI: time between onsets of two consecutive notes) within 10% faster or slower than the expected IOI. We accounted for natural variations in timing by comparing the timing of the expected note to the preceding correct note or to the tempo of the melody if there was no preceding correct note (Wing, 2002). The number of correct IOIs was divided by the length (i.e., the number of IOIs) of the target sequence, or if the performed sequence was longer than the target sequence, by the performed sequence’s length.

Previous work used a more lenient criterion of timing accuracy, considering IOIs 30–50% larger or smaller than expected IOIs as correct (Drake and Palmer, 2000; Brown and Penhune, 2018). According to the previous criterion of 30% faster or slower IOIs, melodies with a tempo of 58–107 bpm would be acceptable renditions of a target tempo of 75 bpm. With our definition, tempi ranging from 68 to 83 bpm were acceptable.

#### Sample Size Calculation

After collecting data from the 25 participants, we ran a sample size calculation to determine the number of participants that would be required to reject the null hypothesis with 80% power. Given the group differences and standard deviations, to reject the null hypothesis with the pitch accuracy data, we would have needed to recruit 396 participants per group, and with the rhythm accuracy data, we would have needed to recruit 156 participants per group.

#### Statistical Analysis

##### Demographics

Independent samples *t*-tests were used to assess group differences in: (a) demographics (age, weight, height), (b) variables that could affect musical learning (auditory working memory, number of extra familiarization trials required, competitiveness, years of formal education, amount of musical training, and amount of piano training), and (c) graded exercise test outcomes (VO_2peak_, W_max_, HR_max_, RER_max_, and RPE_max_).

##### Acquisition

To examine whether both groups had achieved similar performance by the end of the acquisition phase, a Wilcoxon rank sum test was used. This non-parametric test was selected because, for several participants (n = 14), distributions of scores were truncated when they reached criterion performance in the piano acquisition task. To ensure that the melodies did not differ in terms of difficulty, a Wilcoxon rank sum test compared the two melodies on end-of-acquisition pitch and rhythm accuracy. Due to the nature of the acquisition task whereby participants had varying numbers of trials to criterion, the average of the last 10 trials was used to calculate each participant’s end of acquisition performance (Acq).

##### Retention

Non-parametric mixed-effects modeling was conducted using SAS Version 9.4 (SAS Institute Inc., Cary, NC, United States) following the approach of Brunner et al. (2001). Non-parametric mixed-effects modeling is a rank-based method for the analysis of longitudinal experiments and is robust against sampling with unevenly distributed repeated measures, such as in our study design. Previous research examining the effects of exercise on motor learning employed parametric, linear mixed-effects modeling (Thomas et al., 2016a, b, 2017; Dal Maso et al., 2018). However, the dependent measures in the present study did not satisfy the requisite normality, as assessed with a Kolmogorov–Smirnov test. Type III sum of squares was used to test the contribution of the fixed effects. *Post hoc*, model-based comparisons were performed using the least-squares method. All analyses were performed with two-tailed tests and a significance level of *p* = 0.05. Multiple comparison corrections were not performed because of the exploratory nature of the present study.

Separate models were fitted for pitch and rhythm accuracy with fixed effects of group (HIIT, LIIT), session (Acq, R1, R24, R7), group by session interaction, musical training (months, to control for experiential differences), and a random intercept of subject. The data used to build these models can be located in the Supplementary Material.

##### Transfer: Acquisition of a second melody

Separate models were fitted for pitch and rhythm accuracy with fixed effects of group (HIIT, LIIT), block (Blocks 1–6), group by block interaction, musical training (months), and a random intercept of subject.

##### Acquisition and transfer

To examine changes in performance between acquisition and transfer, a model was fitted to blocks 1–3 of acquisition and transfer with fixed effects of group (HIIT, LIIT) by session (Acq, Tran) by block (1–3) interaction, and random intercept of subject in both pitch and rhythm accuracy. Amount of musical training was also included as a fixed effect to control for experiential differences.

## Results

### Demographics and GXT Parameters: No Group Differences

Twenty-five individuals participated in the study (*n* = 25; High = 13, Low = 12). There were no significant differences between the HIIT and LIIT groups on participant characteristics of age [*t*(23) = 0.65, *p* = 0.52], weight [mean ± SD: High = 66.45 ± 10.39 kg, Low = 66.41 ± 13.6 kg, *t*(23) = 0.004, *p* = 0.99], height [mean ± SD: High = 168.2 ± 9.3 cm, Low = 168.6 ± 7.5 cm, *t*(23) = 0.13, *p* = 0.90] competitiveness [*t*(23) = 1.1, *p* = 0.28], and physical activity habits [mean ± SD: High = 1690.5 ± 1553.9 MET-minutes/week, Low = 1796.7 ± 1979.4 MET-minutes/week, *t*(21) = 0.148, *p* = 0.88] (refer to Table 1). No significant differences existed in characteristics that could have influenced musical learning abilities including AWM [*t*(23) = 1.35, *p* = 0.19], number of extra familiarization trials (EFT) [*t*(23) = 0.78 *p* = 0.45], BAT score [*t*(23) = 0.88, *p* = 0.39], years of formal education [*t*(23) = 1.01, *p* = 0.32], amount of musical training [*t*(23) = 0.30, *p* = 0.77], amount of piano training [*t*(23) = 1.14, *p* = 0.27], or amount of sleep and quality of sleep before or after acquisition [amount before: *t*(23) = −0.34, *p* = 0.73; amount after: *t*(23) = 0.95, *p* = 0.35; quality before: *t*(22) = −0.90, *p* = 0.38; quality after: *t*(22) = −1.07, *p* = 0.30]. There were also no significant differences between groups in the graded exercise test (GXT) parameters of VO_2peak_ [*t*(23) = 0.78, *p* = 0.45], maximum power output (W_max_) [*t*(23) = 0.24, *p* = 0.81], maximum heart rate (HR_max_) [*t*(23) = 0.59, *p* = 0.56], maximum respiratory exchange ratio (RER_max_) [*t*(23) = 0.14, *p* = 0.89], or maximum rating of perceived exertion (RPE_max_) [*t*(23) = 0.36, *p* = 0.72].

### Intensity Manipulation and Differences Between Groups in HR and RPE

As expected, there were differences between groups in the interval exercise test, with the HIIT group having a higher HR_max_ [mean ± SD: High = 178.2 ± 11.1, Low = 110.9 ± 12.4, *t*(23) = 14.3, *p* < 0.001] and higher RPE_max_ [mean ± SD: High = 16.9 ± 1.98, Low = 9.5 ± 1.88, *t*(23) = 9.6, *p* < 0.001].

#### Incomplete Interval Exercise Tests

Five participants in the HIIT condition failed to complete the exercise protocol due to volitional exhaustion (participants 16, 17, 20, 21, and 24). According to the American College of Sports Medicine’s (ACSM) fitness categories of maximal aerobic power, all of these participants had very low fitness (Pescatello et al., 2014). After participants 16 and 17 failed to complete the interval exercise protocol, and we observed that these two participants reported that they did not exercise on a weekly basis, anyone who exercised less than once per week was excluded. Despite this additional exclusion criterion, participants 20, 21, and 24 also failed to complete their personalized high-intensity protocol (Table 1). We included data from these participants in all analyses reported below given our “intention-to-treat” approach to analysis (Gupta, 2011). These five participants still exercised at a high intensity as they reported high ratings of perceived exertion (average RPE: 18.4) and exhibited high maximum heart rates (average HR_max_: 184.2) along with those who successfully completed the interval exercise test (average RPE: 16, average HR_max_: 174.5). Therefore, we assumed that they benefited from the physiological effects of the partial HIIT (see Table 2).

**TABLE 2:** Dosage for participants who did not complete the high-intensity interval training.

Participant	Dose of High-Intensity Interval Training
16	10 m 30 s
17	10 m 30 s
20	4 m
21	15 m 45 s
24	11 m

*The majority of participants who did not complete the HIIT exercised until at least the middle of the second high-intensity interval (10 m 30 s).*

### End of Acquisition: No Group Differences

There were no group differences in end-of-acquisition performance for pitch accuracy (*W* = 107, *p* = 0.121) or rhythm accuracy (*W* = 86, *p* = 0.683).

There were also no differences between the two melodies in end-of-acquisition performance for pitch accuracy (*W* = 101, *p* = 0.157) or rhythm accuracy (*W* = 62, *p* = 0.488).

**FIGURE 5:**
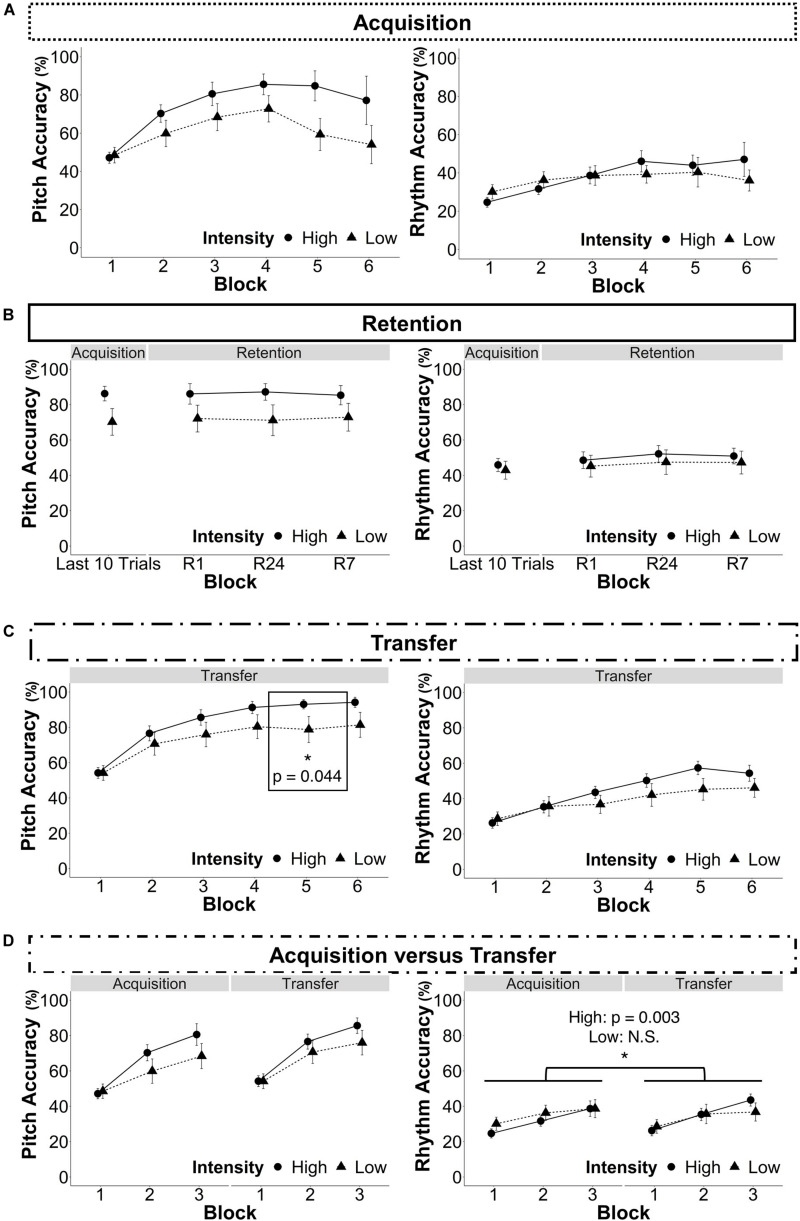
Results. The data points represent the mean and the error bars represent the standard error of the mean. For individual participants’ performance curves, see Supplementary Figure S2 in the Supplementary Material. **(A)** Acquisition (Recall that not all participants completed all trials in blocks 4, 5, and 6 because they practiced until the reached a criterion of performing three consecutive test trials correctly. Therefore there are fewer participants displayed in blocks 4, 5, and 6 than in blocks 1, 2, and 3 (See section “Blocks” for more information). **(B)** Retention. (Last 10 Trials: data from the last ten trials each participant completed, R1: 1-h retention test, R24: 24-h retention test, R7: 7-day retention test). **(C)** Transfer. **(D)** Acquisition vs. transfer. *p < 0.05.

As noted in the statistical analysis plan, only the last 10 trials of each participant’s acquisition were analyzed because of variations in the number of trials to achieve the performance criterion. Nevertheless, Figure 5A shows the full acquisition data. For individual performance curves, refer to Supplementary Figure S2 in the Supplementary Material.

### Retention: No Group Differences

Assumptions of distribution normality for the pitch and rhythm accuracy data were violated (pitch accuracy: *D* = 0.243, *p* < 0.01; rhythm accuracy: *D* = 0.123, *p* < 0.01). Further examination of the data with residual plots revealed that parametric testing on the pitch accuracy data would be inappropriate. Parametric testing would have been robust against slight deviations observed in the residual plots of rhythm, however, for consistency between dependent measures, both models were evaluated with non-parametric methods. All models satisfied the tolerance level of less than 0.4, and no variables were collinear in any model.

For pitch accuracy, there was no significant interaction between group and session, *F*(3,69) = 0.34, *p* = 0.334. Therefore the interaction was removed, and the model was refitted with only the main effects of group, session, and amount of musical training. There were no main effects of group, *F*(1,22) = 2.74, *p* = 0.098, session, *F*(1,72) = 0.51, *p* = 0.674, or musical training, *F*(1,22) = 0.06, *p* = 0.802 (see Figure 5B).

For rhythm accuracy, there was no significant interaction between group and session *F*(3,69) = 0.19, *p* = 0.905, therefore the interaction was removed, and the model was refitted with only the main effects of group, session, and amount of musical training. There were no main effects of group, *F*(1,22) = 0.26, *p* = 0.608 or musical training, *F*(1,22) = 0.53, *p* = 0.465, but there was a main effect of session, *F*(3,72) = 5.95, *p* < 0.001. Model-based, least-squares comparisons revealed that performance improved in both groups from the end of acquisition to R24, *t*(72) = −3.93, *p* < 0.001 and R7, *t*(72) = −3.24, *p* = 0.002.

### Transfer of Learning: Modest Differences Between Groups

For pitch accuracy, there was an interaction between group and block, *F*(5,115) = 6.26, *p* < 0.001, such that the HIIT group had a greater increase over block as compared to the LIIT group (7.9% per block vs. 5.2%). There was a main effect of block, *F*(5,115) = 158.87, *p* < 0.001, but no main effect of group, *F*(1,22) = 1.81, *p* = 0.178, or musical training, *F*(1,22) = 0.40, *p* = 0.528. Model-based least-squares comparisons revealed a significant difference in block 5 where the HIIT group performed better than the LIIT group, *t*(115) = 2.04, *p* = 0.044; HIIT: 93.08 ± 11.20%, LIIT: 78.78 ± 25.84%), but this difference was not maintained in block 6, *t*(115) = 1.82, *p* = 0.072 (see Figure 5C).

For rhythm accuracy, there was an interaction between group and block, *F*(5,115) = 6.94, *p* < 0.001, such that the HIIT group had a greater increase over block as compared to the LIIT group (6.3% per block vs. 3.5%). There was a main effect of block, *F*(5,115) = 74.11, *p* < 0.001, but no main effect of group, *F*(1,22) = 0.82, *p* = 0.366, or musical experience, *F*(1,22) = 0.88, *p* = 0.347. Model-based least-squares comparisons revealed no significant differences, so the interaction may have been driven by a trend that was evident in block 5, *t*(115) = 1.91, *p* = 0.0585.

### Comparing Acquisition and Transfer of Learning: Modest Difference in Rhythm Accuracy

Only blocks 1–3 were analyzed because all participants completed blocks 1–3 in acquisition and transfer (for more information refer to “Blocks” Criterion training). For pitch accuracy, there was a significant interaction between group and block, *F*(2,46) = 15.15, *p* < 0.001, but all other interactions were not significant. Therefore they were removed and the model was re-fit, revealing a main effect of session, *F*(1,24) = 61.46, *p* < 0.001, but no main effect of group, *F*(1,22) = 0.92, *p* = 0.338) or musical experience, *F*(1,22) = 0.26, *p* = 0.610). Model-based least-squares comparisons revealed no significant differences between groups in blocks 1, 2, or 3 (see Figure 5D). To better understand the interaction between group and block, models of acquisition and transfer were built separately. For acquisition alone, there was a significant interaction between intensity and block, *F*(2,46) = 12.10, *p* < 0.001, such that the HIIT group had a greater increase over block as compared to the LIIT group (16.7% per block vs. 9.9%). For transfer alone, there was a significant interaction between intensity and block, *F*(2,46) = 5.76, *p* = 0.003, such that the HIIT group had a greater increase over block as compared to the LIIT group (15.7% per block vs. 10.9%).

**FIGURE 6:**
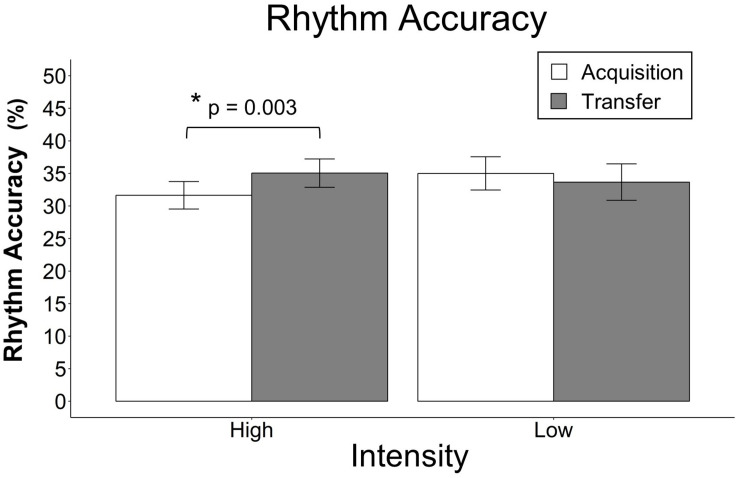
Rhythm accuracy in transfer vs. acquisition. The data points represent the mean and the error bars represent the standard error of the mean. Rhythm accuracy in transfer vs. acquisition averaged across blocks 1–3. The bar graph shows that the HIIT group performed better during transfer than during acquisition and that there was no corresponding difference in the LIIT group. The data from Figure 5D were replotted here to more accurately parallel the statistical analysis that was performed. Note that the scale of this figure is different than Figure 5D.

For rhythm accuracy, there was a significant interaction between group and block, *F*(2,46) = 9.25, *p* < 0.001, and group and session, *F*(1,23) = 10.49, *p* = 0.001, however, the other interactions did not reach significance and were removed. There was a main effect of block, *F*(2,46) = 90.51, *p* < 0.001, but there were no main effects of group, *F*(1,22) = 0.08, *p* = 0.775, or musical experience, *F*(1,22) = 1.82, *p* = 0.1744. Model-based least-squares comparisons revealed that the HIIT group performed better during transfer than during acquisition, *t*(23) = −3.36, *p* = 0.003, but there was no corresponding difference in the LIIT group, *t*(23) = 1.27, *p* = 0.218. Refer to Figure 5D for all data and to Figure 6 for the significant difference. To better understand the interaction between group and block, models of acquisition and transfer were built separately. For acquisition alone, there was a trending interaction between intensity and block, *F*(2,46) = 2.91, *p* = 0.054, such that the HIIT group had a slightly greater increase over block as compared to the LIIT group (7.0% per block vs. 4.3%). For transfer alone, there was a significant interaction between intensity and block, *F*(2,46) = 7.26, *p* < 0.001 such that the HIIT group had a greater increase over block as compared to the LIIT group (8.6% per block vs. 4.1%).

## Discussion

### Summary

In this exploratory study, we asked whether the beneficial effects of HIIT on implicit learning of simple motor tasks (Roig et al., 2012; Mang et al., 2014, 2016b) extend to explicit learning of complex motor tasks such as learning to play piano melodies. To the best of our knowledge, we are the first to examine the effects of HIIT during early consolidation on transfer of motor learning to a new melody. Non-musicians were trained on a piano melody prior to undergoing a personalized bout of high-intensity interval training (experimental group: HIIT) or low-intensity interval training (active control group: LIIT) on a cycle ergometer. Retention was measured 1 h, 1 day, and 1 week after acquisition. There were no group differences at retention, and both groups demonstrated consolidation of rhythm accuracy 1 day and 1 week after the end of the acquisition phase. After the final retention test, participants were trained on a new melody to assess transfer, and the HIIT group demonstrated modestly better transfer than the LIIT group. Although HIIT did not enhance sequence-specific learning on the piano learning task, it had modest benefits on sequence-independent learning (Krakauer et al., 2019).

### Modestly Enhanced Transfer for HIIT Group

Exposure to HIIT after learning one melody modestly improved the acquisition of pitch and rhythmic information in a second melody that was practiced 1 week later. Improved transfer to the rhythm of a new melody was accompanied by improved rate of learning of pitch information. There was also improved pitch accuracy in the fifth block of acquisition of the new melody, but the enhanced performance was not sustained in the sixth block. Accordingly, our results offer tentative support for the possibility that HIIT supports sequence-independent skill learning rather than sequence-specific learning in the context of piano playing (Seidler, 2010; Krakauer et al., 2019).

Sequence-independent learning is important because it supports the learning of new information (Krakauer et al., 2019). Enhancing task-general transfer is particularly important for music students who must learn a repertoire consisting of many pieces. Improving transfer of learning increases learner productivity.

Because HIIT reduces cerebellar inhibition, *trans-*cerebellar pathways are thought to mediate exercise-induced increases of M1 plasticity (Mang et al., 2014, 2016a), and transfer of learning is related to increased dependence on the cerebellum (Seidler, 2010), it is possible that the HIIT group’s improved transfer is attributable to changes in cerebellar activity. Furthermore, rhythmic processing and musical performance also rely on the cerebellum (Penhune et al., 1998; Zatorre et al., 2007). Future research could combine behavioral and neuroscientific methods to determine if cerebellar activity underlies the exercise-induced enhancements in transfer of learning. An alternative explanation is that exercise makes memories more flexible, allowing them to be reconsolidated more easily, possibly via enhanced hippocampal functioning (Siette et al., 2014). Thus, it is possible that HIIT permitted enhanced modification of the first learned sequence to promote learning the second sequence.

### Learning Observed in Both HIIT and LIIT Groups: No Effect of Exercise Intensity on Retention

Both the HIIT and LIIT groups demonstrated rhythm consolidation, with improved performance 1 day and 1 week after acquisition. The implication is that sequence-specific rhythmic learning is consolidated regardless of exercise intensity. For pitch accuracy, there were no differences between the end of acquisition and the retention tests, which implies that skill levels were maintained.

In previous research, exercise generated enhancements in motor performance at retention relative to the end of acquisition for several implicit motor sequence learning tasks (Roig et al., 2012; Skriver et al., 2014; Mang et al., 2016b; Thomas et al., 2016b, 2017), but it failed to enhance motor learning of visuomotor adaptation tasks (Ferrer-Uris et al., 2017), complex laparoscopic skills (Chartrand et al., 2015), and locomotion (Charalambous et al., 2018).

### Possible Explanations for Lack of Retention Effects

There are several possible explanations for our failure to find effects of exercise on motor retention. Differences between the results of the present study and previous research may be attributable to study design (an active control group in the present study), participant characteristics (fitness, sex, and musical abilities), and task characteristics (explicit vs. implicit task, task complexity). Additionally, it is possible that the effects of exercise on motor learning are very small or specific to the simple tasks used in previous research (Roig et al., 2012). Alternatively, HIIT may not enhance the retention of motor sequences, given that another study failed to replicate the retention effects (Hung et al., 2019).

#### Active Control Group

Previous research on the effects of exercise on motor learning used resting control groups in which participants sat in silence and/or read material. Because enhancements are thought to be driven by HIIT, a low-intensity exercise group seemed like a more appropriate control group for the present study, as even moderate intensity exercise produces smaller benefits than HIIT (Thomas et al., 2016b). Because small, positive benefits have been observed for moderate-intensity interval training, we devised a very low-intensity interval exercise protocol (Thomas et al., 2016b). It is possible, however, that our low-intensity exercise was insufficiently low. For participants naïve to cycling, maintaining a cadence of 70–90 RPM may have been physiologically challenging despite the low power output. Future research could consider including a resting control group in addition to an active control group with a self-selected cadence. However, if differences are observed between an experimental group and resting control group, it is possible that these differences can be attributed to differences in arousal and not the biological effects of HIIT.

The mean RPE of the LIIT group was 9.5 during the interval exercise test, which was lower than the HIIT group’s mean of 17 but higher than that experienced by a person not engaging in any activity: 6 (Borg and Kaijser, 2006). The average maximum heart rate in the LIIT group was 111 ± 12 bpm, as compared with 178 ± 11 bpm for the HIIT group. Although the lower exercise intensity may not have caused the release of neurochemicals that are believed to underlie enhancements of exercise on motor learning, the physical activity may have resulted in improved blood flow or arousal. Other research with a mild exercise protocol of 10 min at 30% VO_2peak_ with a cycling cadence of 60 RPM found an average RPE of 11 ± 2.3 and average HR of 107 ± 9 bpm (Byun et al., 2014). The authors observed improved arousal level and cognition with this acute mild exercise.

#### Participant Characteristics

##### Fitness

The fitness of participants in the present study was much lower than those reported in previous studies. The average VO_2__peak_ was 30.5 ± 8 ml/kg/min for the high-intensity group, and 33.1 ± 8.5 ml/kg/min for the low-intensity group. In previous research, participants exhibited an average VO_2__peak_ ranging from 43 to 45 ml/kg/min (Mang et al., 2016a). Perhaps some minimal fitness level is required for observable benefits of exercise on motor learning. In fact, one meta-analysis revealed a positive association between fitness measured through VO_2__peak_ and BDNF release after acute exercise (Dinoff et al., 2017). On the basis of this meta-analysis, the predicted standardized mean difference in BDNF release of our sample would be approximately 0.3 compared to approximately 0.8 predicted based on the fitness of participants in other studies. If BDNF underlies the effects of exercise on motor learning, then the majority of participants in the present study would not have benefited from the HIIT intervention. However, one study that examined fitness level as a mediator of the effects on motor learning failed to observe exercise-induced enhancements in a 24-h retention test for high and low fitness groups (Hung et al., 2019).

Several participants (participants 4, 6, 8, and 14) had lower HRs at the end of the graded exercise test than would be expected if they had truly reached their cardiovascular maximum. The volitional exhaustion of these participants may not have been caused by maximal cardiovascular exertion but rather because their muscles could not produce the required increase in power output, resulting in an inaccurate measure of their VO_2__peak_. Future exercise test protocols could use continuous ramping GXT protocols on samples that are not trained athletes (Arena et al., 2007).

##### Sex

The meta-analysis that reported an association between fitness and BDNF release also revealed an association between sex and BDNF release (Dinoff et al., 2017). Specifically, the greater the percentage of male participants in a study, the higher the standardized mean difference. Our participants were 40% male, as opposed to other studies with males only (e.g., Roig et al., 2012), which may have contributed to our small effect size.

##### Musical abilities

Several participants failed to learn the melodies within the time constraints of the acquisition protocol. Musical aptitude varies greatly across individuals, with one study reporting that non-musicians required 12–70 min to learn a 15-note melody by ear (Lahav et al., 2007). Because musical aptitude affects the likelihood of pursuing musical training (Swaminathan et al., 2017), our inclusion criterion of little or no musical training may have resulted in participants with relatively low musical aptitude. Even if exercise enhances motor consolidation, it may be of limited value for participants who cannot acquire the motor sequence, perhaps even consolidating incorrect motor sequences. Future research could examine whether exercise promotes learning in non-musicians with moderate musical aptitude, as indicated by a musical aptitude test (e.g., Gordon, 1965), and in musicians.

In contrast, several other participants readily learned the melody and maintained their pitch accuracy, resulting in ceiling effects that precluded the possibility of observing exercise-induced enhancements. Future research on the explicit learning of piano or other motor sequences could employ longer retention intervals, generating more forgetting and greater opportunity of observing savings afforded by exercise.

#### Task Characteristics

##### Explicit motor learning

Previous research on exercise and sequence learning examined implicit learning of motor sequences (Mang et al., 2014, 2016b). The explicit learning of motor sequences, as in the present study, may rely on different neural systems for consolidation (Robertson et al., 2004; Robertson, 2009; Song, 2009). Specifically, implicit consolidation relies on the primary motor cortex while explicit consolidation relies on the dorsal premotor cortex and supplementary motor area (Honda et al., 1998; Kantak et al., 2012). Exercise may enhance implicit motor consolidation through its ability to reduce inhibition or increase excitation of the primary motor cortex (Coco et al., 2016; Neva et al., 2017). However, excitability of the supplementary motor area is decreased after exhaustive exercise (Coco et al., 2016). It is possible, then, that the null results in the present study stemmed from the failure of exercise to promote enhanced consolidation of explicit motor sequence learning.

##### Complexity

The piano learning task developed for this study involved reduced feedback as participants progressed to facilitate memorization of the sequence. The present task may have required greater cognitive effort than tasks in other studies (e.g., visuomotor tracking, SRTT) because of the requirements of memorization and timing. Because cognitive effort enhances motor learning (Lee et al., 1994), the ceiling effects observed at 1-week retention may be attributable to task characteristics rather than musical aptitude. Perhaps future research with motor tasks that involve larger cognitive contributions could use longer retention intervals to more readily detect memory savings afforded by exercise.

### Limitations

There are a number of graded exercise test and HIIT protocols that have been implemented in previous research. We selected exercise protocols that had previously produced positive findings when examining the influence of HIIT on motor learning. This allowed us to maintain consistency between the exercise parameters of the previous research and the current research which enables a more direct comparison between the current study and previous research. An examination of how the exercise protocols may be improved is important for future research.

The ACSM provides gold standard recommendations for exercise testing. The ninth edition of the ACSM guidelines suggest than that deconditioned individuals could do a graded exercise test on a cycle ergometer with 10–15 W increases every minute (American College of Sports Medicine [ACSM], 2014). This is similar to the 30 W increments every 2 min employed in our study. Furthermore, the ACSM guidelines (2014) recommend that graded exercise test protocols should be personalized based on age and fitness, therefore the sedentary participants could start at a lower starting workload. The ninth edition of the ACSM guidelines makes no recommendation on cycling cadence, however, one study found that deconditioned non-cyclists preferred a higher cadence with lower loads (80 RPM for 75 W), but a lower cadence for higher loads (65 RPM for 175 W) therefore perhaps self-selected cadence could be used for participants lacking cycling experience (Marsh and Martin, 1996). Training experience can also facilitate greater recovery between high-intensity intervals, therefore this should also be considered when prescribing the low-intensity workload (Messonnier et al., 2013). Future research should match the exercise protocols more closely to the characteristics of their sample, such as the frequency and intensity of their exercise and their cycling experience.

Five participants failed to complete the full HIIT exercise protocol due to exhaustion. These participants still had high ratings of perceived exertion and high HR_max_, much like those who completed the HIIT protocol. However, it is possible that their shorter HIIT dosage failed to improve their motor consolidation, though their learning curves do not appear to show this (see Supplementary Figure S2). While these participants reached exhaustion, it is possible that they did not experience the same increases in neurochemicals and lactate as that provided by completing the full protocol. Future research could recruit participants who exercise regularly unless they use a HIIT protocol with lower active rest intensities that may be suitable for relatively sedentary participants (e.g., Kriel et al., 2016). Alternatively, the interval exercise protocol could be prescribed based on maintaining target HRs, percent of HR reserve, or based on measures of gas exchange instead of work intensities to ensure that the workload is both not too challenging for deconditioned individuals and matched across participants in relation to their anaerobic thresholds (Mann et al., 2013; Singh et al., 2016; Stavrinos and Coxon, 2017). However, one methodological review suggests that prescribing intensities based on percentage of HR_max_, HR reserve, VO_2max_, or VO_2_ reserve can cause considerable interindividual differences in cardiopulmonary, metabolic, and physiological strain because of differences in the HR-performance curve and differences in VO_2max_ performance (Tschakert and Hofmann, 2013). The authors state that the usage of %VO_2max_ and %HR_max_ is inappropriate especially for high-intensity intervals with long durations, such as that in the present study, due to the heterogeneity of acute physiological responses, which increases with the duration of the interval. They recommend using submaximal thresholds or turn points based on gas exchange as well as the W_max_. Since we wanted to maintain consistency between the other studies in this field to improve our ability to compare results, and because this was a prescription method recommended in a methodological review, we believed that it would be best to use W_max_ during prescription (Tschakert and Hofmann, 2013). However, it is possible that this prescription method caused excessive strain on the five participants who were unable to complete the test, causing sample attrition. Other prescription methods could be explored in future research.

Turning attention to the motor learning task, the dependent measures could be improved. In the present study, pitch and rhythm accuracy were scored as percentages rather than absolute values, yielding a maximum possible score of 100%. Participants were trained to a criterion of 100% and several demonstrated maintenance of learning at retention. As a result, potential enhancements of exercise may have been obscured by the truncation effect of participants at ceiling performance levels. More sensitive measures are necessary as well as longer retention intervals after acquisition. Pilot testing can ascertain appropriate levels of task difficulty and retention intervals that promote some degree of forgetting.

There was considerable variability in skill acquisition, which may be attributable to musical aptitude, ability, or motor learning ability in general (see Supplementary Figures S1, S2). Although the analysis with random intercept of subject accounts for each individual’s initial performance, large variability in rate of learning can still obscure potential effects of exercise. Given the high variability in participants’ ability to learn a piano melody, the current sample was underpowered to detect the desired effects. A sample size test with the between-group differences in retention revealed that a substantially larger sample size (∼300/group) would be required to observe a significant difference. When examining the sample size calculation for transfer and the modest effects of exercise on transfer of learning, the effects of acute HIIT appear to be small for piano learning.

Nonetheless, the recruitment of non-musicians should maximize the likelihood of observing any real effect. The question remains as to whether musicians might benefit from a HIIT intervention. Expert performers could reach ceiling effects quickly occluding the possibility of detecting differences unless the intervention was timed to assist musicians in learning a new piece or a particularly challenging passage. Two populations of interest for future research are expert musicians and individuals who are just beginning to practice music because these participants may have sufficient musical aptitude to benefit from improved motor consolidation.

Whether the modest difference between groups is truly meaningful warrants consideration. There was a 3% difference in the rate of learning of pitch information between the high-intensity and low-intensity groups. This 3% difference in rate of learning resulted in a statistically significant 14% difference at block 5, which was not sustained into block 6. The participants in this study exercised for one 20-min bout, but future research could implement HIIT training over a longer period to examine whether the increased learning rate is sustained across multiple bouts. While the participants in the present study only practiced for 20-min, professional musicians and students practice for longer durations and more frequently. If this small effect of improved rate continues over longer periods, it could lead to meaningful differences and be relevant to music practitioners. Future research could also examine musicians’ subjective satisfaction because HIIT can be unpleasant so it remains for individuals to determine whether the benefits of the exercise are worth the discomfort.

It was not possible to blind the experimenter to the group assignment. Therefore, this study was single-blind such that the participants were unaware that there were two groups and participants were blind to the hypothesis. Because group assignment occurred after participants began the acquisition phase, it is unlikely that experimenter effects occurred prior to or during the acquisition phase. However, it is still possible that there were experimenter effects during the interval training, retention, and transfer.

Due to the small sample size and small difference between groups in transfer, the possibility of a type 1 error cannot be ruled out until future research can corroborate the findings. This exploratory research aimed to build a foundation for future work. Future research should recruit a larger sample size to increase statistical power and confirm or reject the findings of this exploratory study.

## Conclusion

The present exploratory study examined the effects of an acute bout of HIIT on the consolidation of explicit motor sequence learning involved in learning to play a piano melody. Contrary to our hypothesis, HIIT did not enhance consolidation and subsequent retention of a piano melody. Instead, HIIT and LIIT groups maintained their performance levels between the retention test 1 h after learning and the retention test 1 day and 1 week later. However, HIIT promoted modest transfer of learning. HIIT may affect sequence-independent learning, but more research is required to examine the effect with higher power and its generalizability to other populations and tasks.

## Data Availability Statement

All datasets presented in this study are included in the article/Supplementary Material.

## Ethics Statement

The studies involving human participants were reviewed and approved by the University of Toronto Research Oversight and Compliance Office – Human Research Ethics Program. The participants provided their written informed consent to participate in this study.

## Author Contributions

DS and JC designed the experiment and wrote the manuscript. DS collected and processed the data. AK, JC, and DS conducted statistical analyses. ST, LT, DA, AK, JC, and DS edited and approved the final manuscript. All authors contributed to the article and approved the submitted version.

## Conflict of Interest

The authors declare that the research was conducted in the absence of any commercial or financial relationships that could be construed as a potential conflict of interest.

## Footnotes

1 www.python.org

2 https://mido.readthedocs.io/en/latest/

3 https://pyautogui.readthedocs.io/en/latest/

4 https://pypi.python.org/pypi/dill

## Abbreviations

BDNF: brain-derived neurotrophic factor
HIIT: high-intensity interval training
HR: heart rate
LIIT: low-intensity interval training
VO_2peak_: peak oxygen uptake
W_max_: maximum power output.
