# Supplementary material for: HIIT the Road Jack: An Exploratory Study on the Effects of an Acute Bout of Cardiovascular High-Intensity Interval Training on Piano Learning
Source: Front Psychol. 2020 Sep 10;11:2154. doi: 10.3389/fpsyg.2020.02154 (PMC7511539; doi:10.3389/fpsyg.2020.02154)
Supplement: Supplementary file 1 [file Data_Sheet_1.pdf]

Supplementary Material

HIIT the road Jack: Aerobic high-intensity exercise has modest benefits for piano learning

Dana Swarbrick*, Alex Kiss, Sandra Trehub, Luc Tremblay, David Alter, Joyce L Chen

*** Correspondence:** Corresponding Author: dana.swarbrick@imv.uio.no

Supplementary Data

Data that were included in the non-parametric mixed effects modelling analysis have been provided in the file named Table 1.xlsx.

Column Descriptions:
Participant: 1-25 corresponding to the participant number

ModelNumber: 1-5 corresponding to the model (Model 1: Acquisition; Model 2: Retention & Last 10 Trials of Acquisition; Model 3: Transfer; Model 4: Blocks 1-3 of Acquisition & Transfer)

SessionType: Acquisition: first piano melody training session. Participants stopped training after they had successfully performed the note sequence 3 consecutive times; Retention: test of the piano melody 1 hour, 24 hours, and 7 days later; Transfer: learning a new melody

SessionNum: 1: Acquisition; 2: 1-hour Retention; 3: 24-hour Retention; 4: 7-day Retention; 5: Transfer

Block: Trials were grouped into blocks of 5 trials per block. Acquisition and transfer both have 6 blocks in total though some participants only completed blocks 1-3 in acquisition because of the training criterion. Once participants performed the pitch sequence correctly 3 consecutive times, they moved on to the interval exercise protocol.

TestNumber: Corresponds to the trial number. There were 10 trials in each retention test. There were between 16 and 30 trials in the acquisition session. Every participant completed 30 trials in the transfer session.

Intensity: Corresponds to the intensity of exercise the participant was prescribed for the interval exercise test.

Melody: Corresponds to the melody the participants learned. If they learned melody 1 during acquisition, they learned melody 2 during transfer, and vice versa.

Pitch_Accuracy & Rhythm_Accuracy: Pitch and rhythm score percentages from that trial

MonthsLearningMusic: The number of months the participant had previously spent learning any musical instrument or voice throughout their life including in school

SUPPLEMENTARY FIGURE 1|(A) Individual participants' pitch accuracy (%) and (B) rhythm accuracy (%) during test trials across the entire experiment. Data represents the block's mean and the error bars represent standard error of the mean.

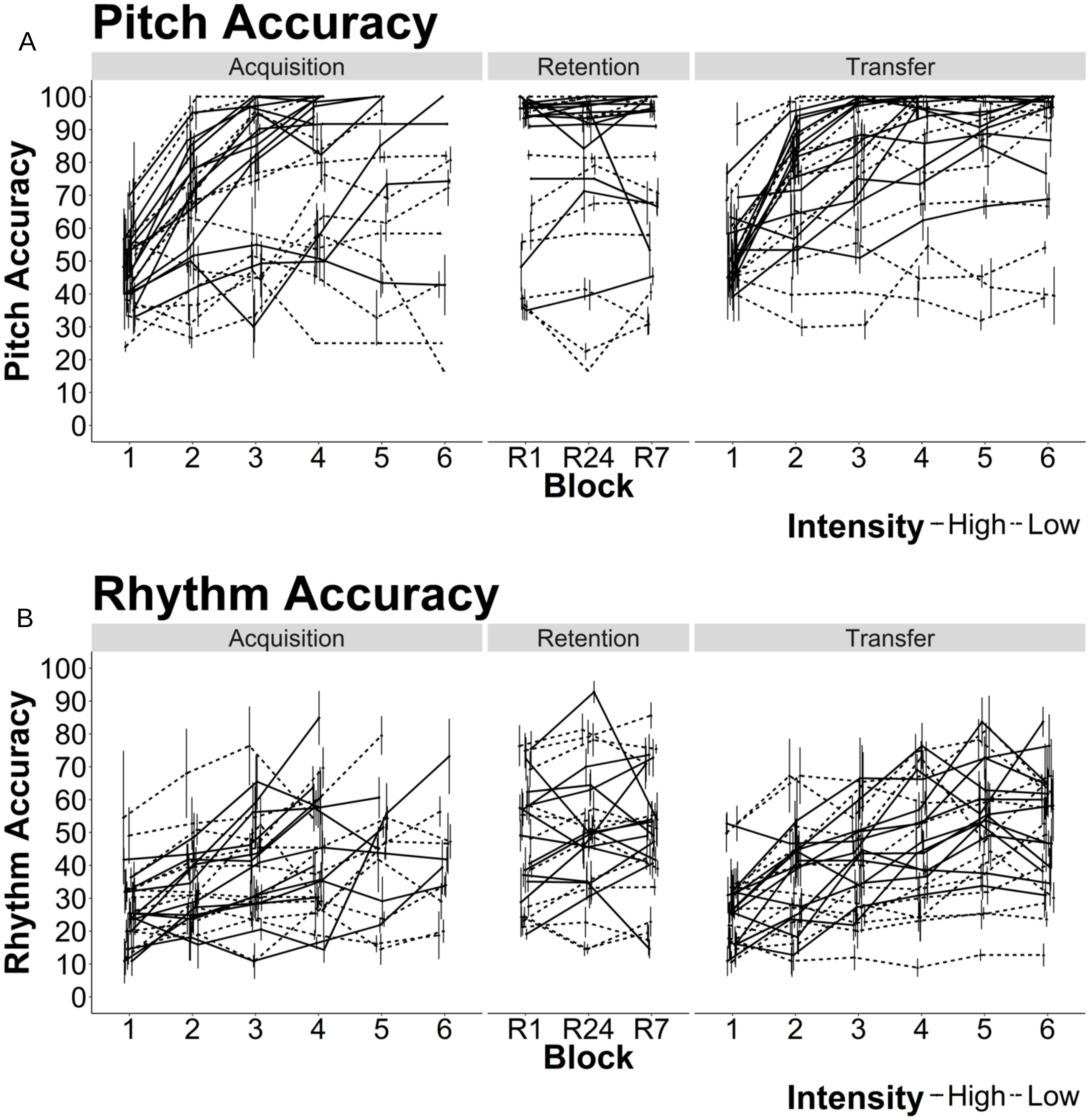

SUPPLEMENTARY FIGURE 2 | Individual participants' performance curves in pitch and rhythm accuracy (%) from test trials during acquisition, retention, and transfer broken down into the high-intensity group (A) and low-intensity group (B). Data represents the block's mean and error bars represent standard error of the mean.

A

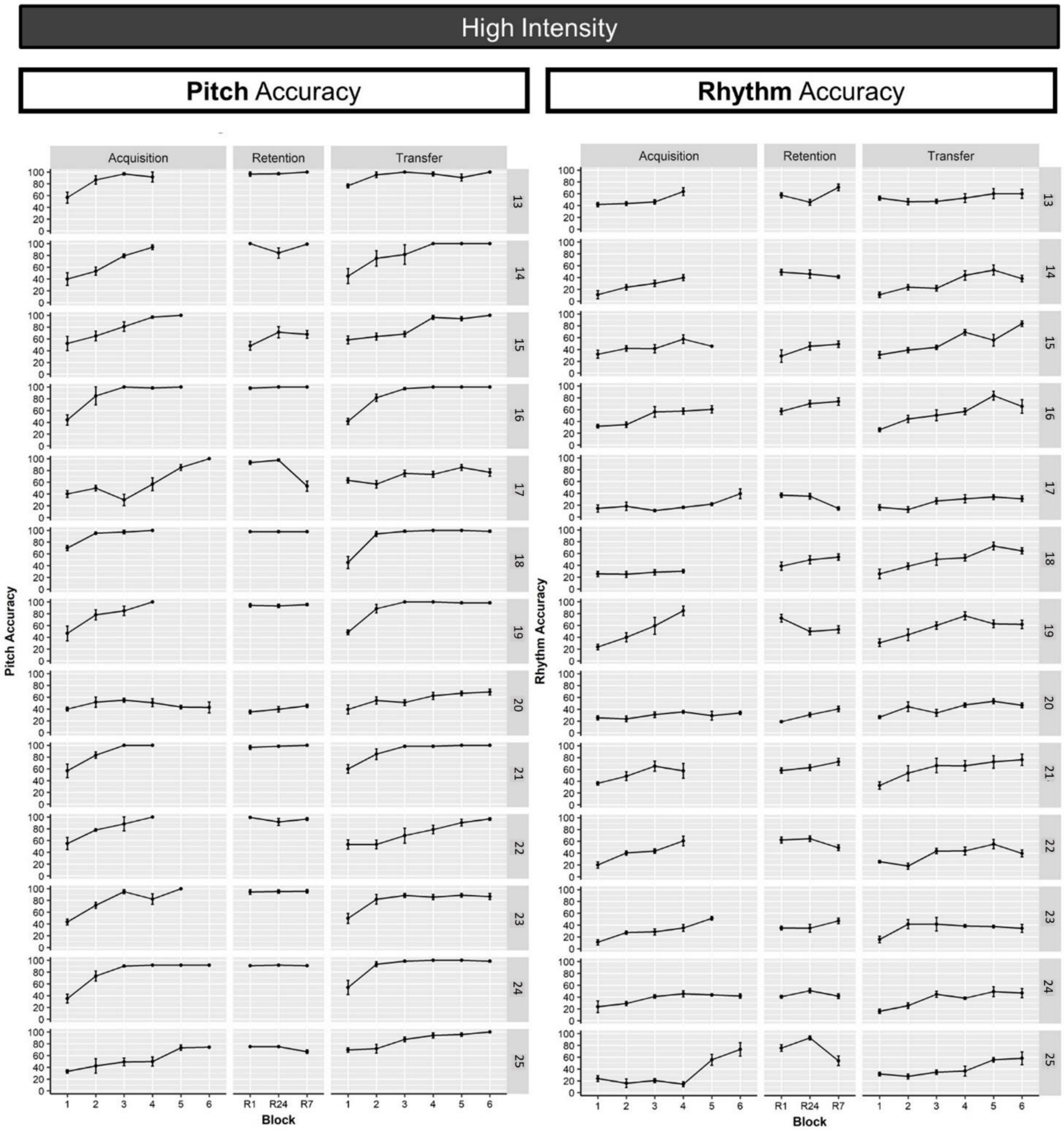

B

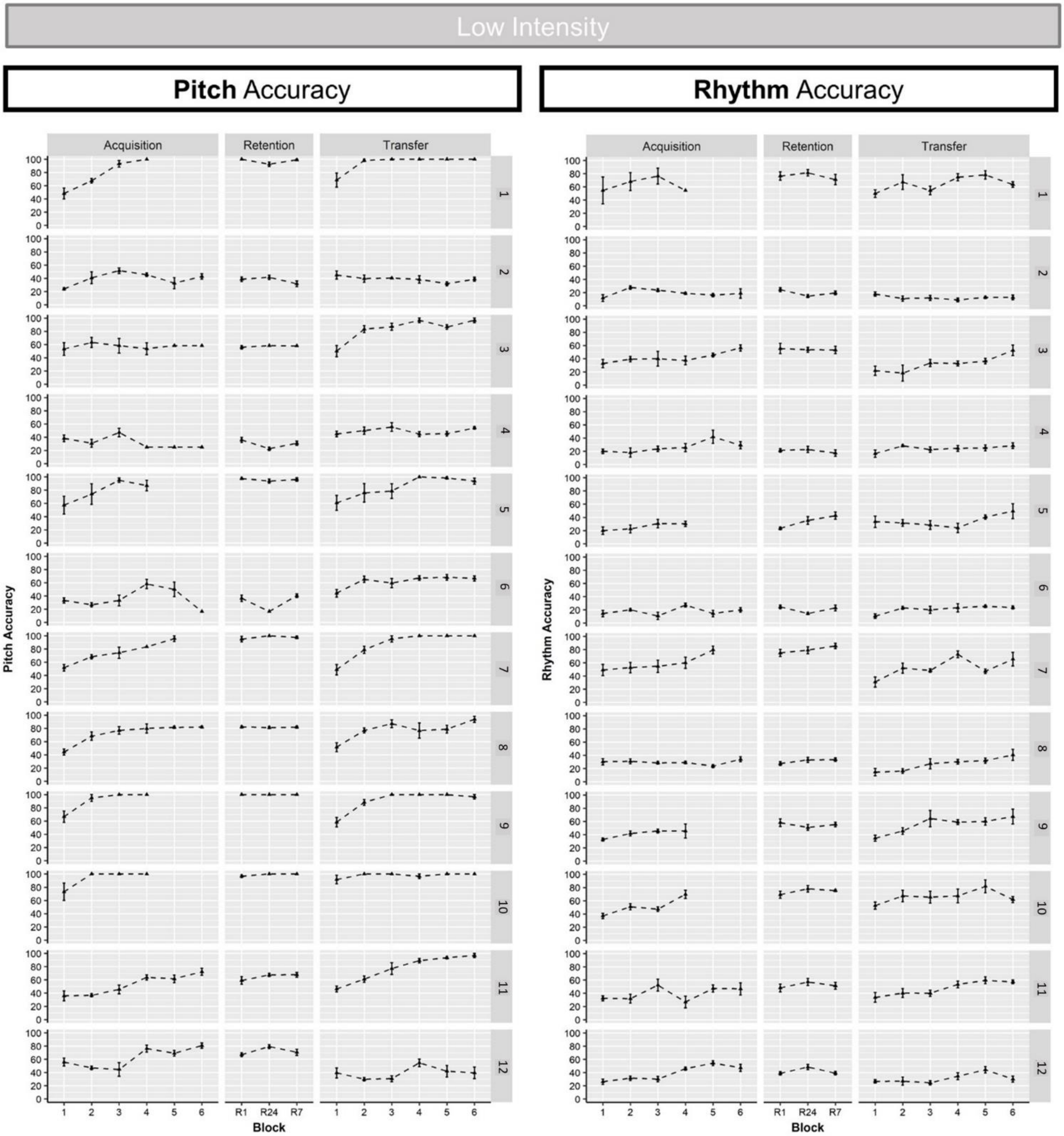
Low Intensity
Pitch Accuracy
Rhythm Accuracy
Acquisition
Retention
Transfer
Pitch Accuracy
Rhythm Accuracy
Block
R1
R24
R7

SUPPLEMENTARY TABLE 1| Sleep Duration and Quality. Participants reported the duration (hours) and quality (on a 7-point scale where 7 is the highest) of their sleep before and after the acquisition session and interval exercise training. Shaded participants are the participants who did not complete the HIIT protocol.

Participant	Intensity	Duration of sleep before acquisition	Quality of sleep before acquisition	Duration of sleep after acquisition	Quality of sleep after acquisition
1	Low	7.5	6	7	5
2	Low	8	5	8	7
3	Low	6	6	9.5	7
4	Low	5	7	4	5
5	Low	8	5	7	6
6	Low	8.5	6	9	5
7	Low	7	5	7.5	5
8	Low	6.5	3	6	1
9	Low	5.25	4.5	6	4
10	Low	5	3	6	4
11	Low	6	5	6	5
12	Low	5	4	9	4
13	High	7.5	5	7.5	6
14	High	8	6	5	6
15	High	8	7	6	5
16	High	6	5	7.5	5
17	High	6.5	5	7	5
18	High	8	7	9	7
19	High	4.5	1	6	5
20	High	8	6	7.5	7
21	High	5	7	7	7
22	High	7	6	6.5	5
23	High	6	6	8	6
24	High	5	5	4	5
25	High	7	5	3	2
